# Supplementary material for: Segmental analysis by speckle-tracking echocardiography of the left ventricle response to isoproterenol in male and female mice
Source: PeerJ. 2021 Mar 12;9:e11085. doi: 10.7717/peerj.11085 (PMC7958899; doi:10.7717/peerj.11085)
Supplement: Table S1 — BW: body weight, LV: left ventricle, EDD: end-diastolic LV diameter, ESD: end-systolic diameter, IVS: inter-ventricular septum, PW: posterior wall, RWT: relative wall thickness, FS: fractional shortening, EDV: end-diastolic volume, ESV: end-systolic volume, SV: stroke volume, EF: ejection fraction, HR: heart rate, CO: cardiac output, IVRT: isovolumetric relaxation time. Values are expressed as the mean +/- SEM. Control and Iso group comparisons were made using Student’s T-test. [file peerj-09-11085-s001.docx]

**Table S1. Left ventricle morphology and function in male and female mice at baseline**. BW: body weight, LV: left ventricle, EDD: end-diastolic LV diameter, ESD: end-systolic diameter, IVS: inter-ventricular septum, PW: posterior wall, RWT: relative wall thickness, FS: fractional shortening, EDV: end-diastolic volume, ESV: end-systolic volume, SV: stroke volume, EF: ejection fraction, HR: heart rate, CO: cardiac output, IVRT: isovolumetric relaxation time. Values are expressed as the mean +/- SEM. Control and Iso group comparisons were made using Student's T-test.

| Parameters | Males (n=18) | Females (n=18) | P value |
| --- | --- | --- | --- |
|  |  |  |  |
| Body weight, g | 24.6 ± 0.31 | 18.4 ± 0.26 | <0.0001 |
| Tibial length, mm | 20.5 ± 0.05 | 19.8 ± 0.03 | <0.0001 |
|  |  |  |  |
| EDD, mm | 3.80 ± 0.03 | 3.55 ± 0.04 | <0.0001 |
| ESD, mm | 2.55 ± 0.05 | 2.26 ± 0.06 | 0.0013 |
| IVS, mm | 0.80 ± 0.01 | 0.77 ± 0.02 | 0.20 |
| PW, mm | 0.82 ± 0.01 | 0.76 ± 0.01 | 0.0031 |
| RWT | 0.43 ± 0.01 | 0.43 ± 0.01 | 0.73 |
| FS, % | 33.0 ± 1.03 | 36.5 ± 1.33 | 0.052 |
| LV mass, mg | 88 ± 3.0 | 73 ± 3.0 | <0.0001 |
|  |  |  |  |
| EDV, µl | 51.6 ± 1.63 | 42.5 ± 1.58 | 0.00033 |
| ESV, µl | 20.8 ± 0.90 | 16.2 ± 0.71 | 0.00029 |
| SV, µl | 30.8 ± 1.27 | 26.3 ± 0.94 | 0.0081 |
| EF, % | 59.6 ± 1.35 | 62.1 ± 0.59 | 0.11 |
| HR, min^-1^ | 471 ± 10.0 | 454 ± 10.2 | 0.24 |
| CO, ml/min | 14.6 ± 0.83 | 11.9 ± 0.45 | 0.0081 |
|  |  |  |  |
| E wave, mm/s | 651 ± 18.6 | 603 ± 13.4 | 0.044 |
| A wave, mm/s | 434 ± 15.7 | 401 ± 10.1 | 0.092 |
| E/A | 1.51 ± 0.03 | 1.51 ± 0.02 | 0.89 |
| E wave decel. time, ms | 19.6 ± 0.64 | 18.7 ± 0.82 | 0.40 |
| E’ mm/s | 27.0 ± 0.69 | 26.2 ± 1.11 | 0.55 |
| E/E’ | 24.4 ± 0.98 | 23.5 ± 0.82 | 0.50 |
| A’ mm/s | 17.3 ± 0.47 | 17.2 ± 0.82 | 0.93 |
| E’/A’ | 1.56 ± 0.03 | 1.53 ± 0.03 | 0.42 |
| IVRT, ms | 15.1 ± 0.30 | 17.7 ± 0.41 | <0.0001 |
