# Supplementary figures and images for: Segmental analysis by speckle-tracking echocardiography of the left ventricle response to isoproterenol in male and female mice
Source: PeerJ. 2021 Mar 12;9:e11085. doi: 10.7717/peerj.11085 (PMC7958899; doi:10.7717/peerj.11085)

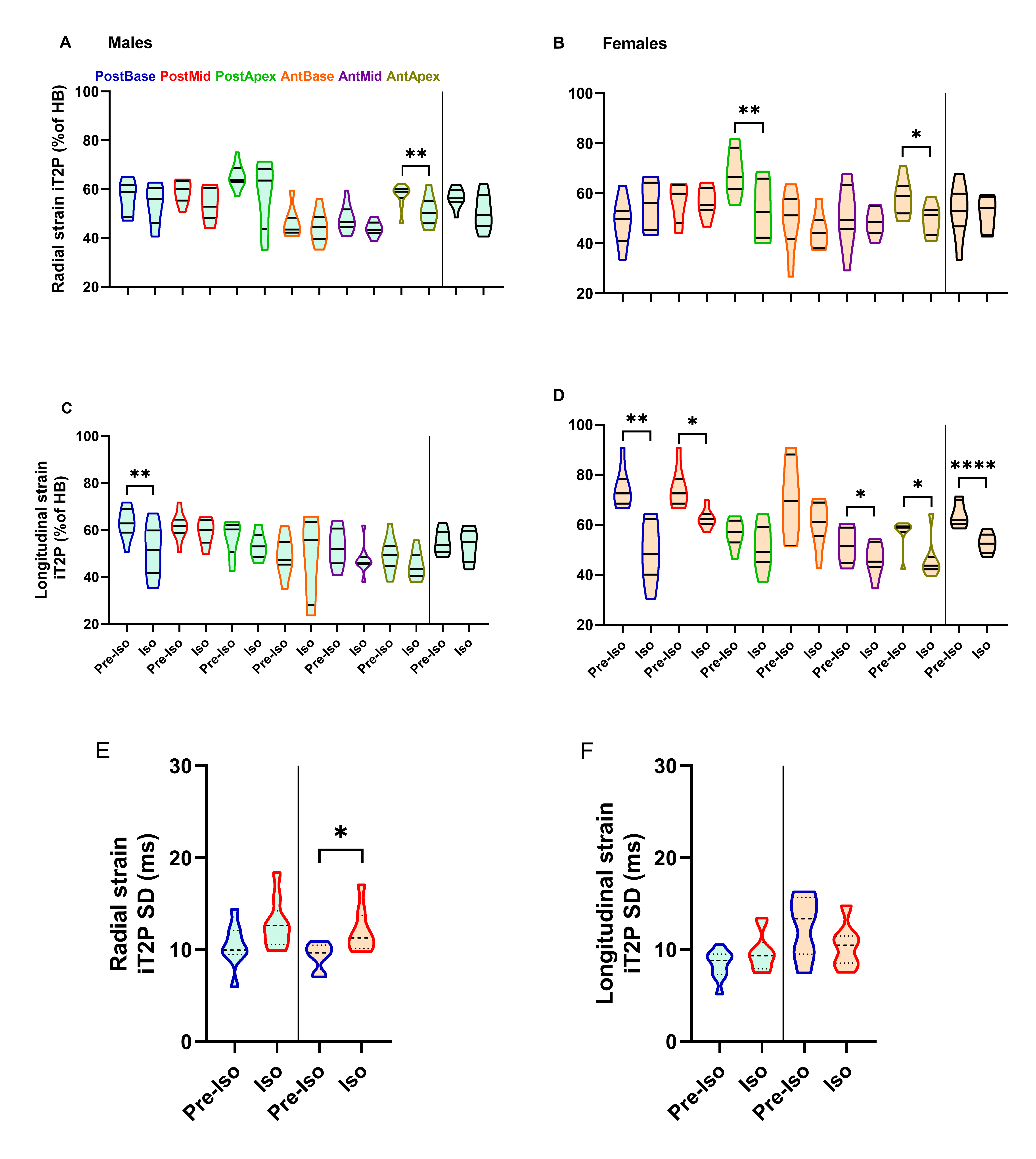

Supplement: Figure S1 — Radial (A–B) and longitudinal (C–D) iT2P were obtained using the parasternal long axis view. The six segments are identified. Characters colors correspond to those used in the graphs for each of these LV segments. Radial (E) and longitudinal (F) iT2P standard deviation between the six segments were calculated for each individual within each group (blue) and females (orange). Results are represented as violin plots (n = 8–10). Significance between groups was calculated with paired Student’s T-test. *: p < 0.05, **: p < 0.01 and ****: p < 0.0001 between corresponding pre-Iso and Iso animals. [file peerj-09-11085-s002.png]

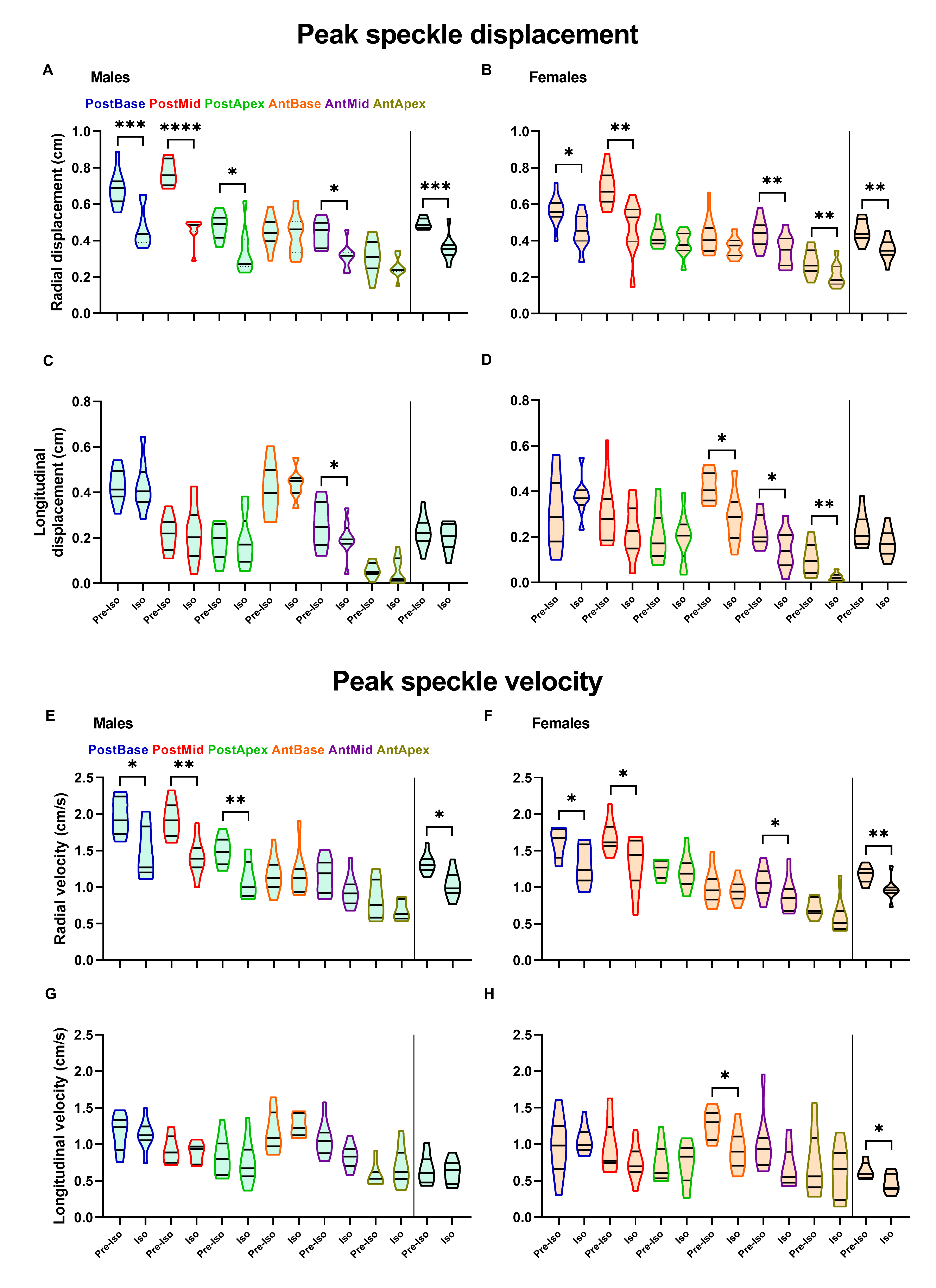

Supplement: Figure S2 — Ant: anterior, Post: posterior, SW: septal wall and FW: free wall. Males are represented on the left panels and females on the right. Results are represented as violin plots (n=8-10). Inner black lines represent quartiles of the data. Significance between groups was calculated with paired Student’s T-test. *: p < 0.05, **: p < 0.01 and ***: p < 0.001 between corresponding pre-Iso and Iso animals. [file peerj-09-11085-s005.png]
